# Telemedicine preferences in pediatric urology following the COVID-19 pandemic: A caregiver survey

**DOI:** 10.3389/fruro.2023.994540

**Published:** 2023-02-13

**Authors:** Tara Bates, Beverly Spray, Stephen Canon

**Affiliations:** ^1^ University of Arkansas for Medical Sciences, Little Rock, AR, United States; ^2^ Arkansas Children’s Research Institute (ACRI), Little Rock, AR, United States; ^3^ Pediatric Urology, Arkansas Children’s Hospital, Little Rock, AR, United States

**Keywords:** telemedicine, pediatric urology, COVID - 19, post COVID, telehealth, pediatrics

## Abstract

**Purpose:**

The COVID-19 pandemic dramatically changed the way many patients interacted with their healthcare providers, with many people being forced to use telemedicine out of necessity. Our study aimed to investigate if this increased usage of telemedicine impacted pediatric patient caregivers’ perception of telemedicine for pediatric urology visits.

**Materials and Methods:**

A prospective survey was administered to the primary caregiver of all patients less than 18 years of age during either an in-person (IP) or a telemedicine (TM) encounter. The survey included questions regarding accessibility to and opinions toward telemedicine.

**Results:**

Two hundred, thirty-nine total patient caregivers were surveyed: 209 IP and 30 TM. Most caregivers in both cohorts reported being more likely to use telemedicine now than before the pandemic: IP (125/209, 59.8%) and TM (23/30, 76.7%). Caregivers also reported that the severity of their child’s condition would impact their likelihood to utilize telemedicine for evaluation (IP 162/209 (77.5%) vs. TM 28/30 (93.3%) with caregivers in the TM group even more likely to be influenced by this factor (p = 0.045). Most caregivers in both groups reported that they would utilize telemedicine within 60 miles from the provider. Over 80% of families from both groups reported having both a laptop and a cellular phone in their home. A greater percentage of caregivers in the IP group reported having a desktop computer and a tablet in their home compared to the TM group (41.1% versus 20.0% and 27.3% versus 3.3%, respectively).

**Conclusions:**

Living through the COVID-19 pandemic has increased the likelihood of caregivers to utilize telemedicine for care of their child’s pediatric urologic disorder. Factors such as severity of illness, distance from the provider, and the context of the evaluation influenced caregiver preferences for utilization of telemedicine. All families surveyed reported having a device at home to perform telemedicine. Laptops and cellular phones were the most commonly used devices.

## Introduction

The COVID-19 pandemic forced many daily activities, including healthcare appointments, to be performed remotely *via* telemedicine due to recommended quarantine and distancing guidelines. This led to a dramatically increased demand for telemedicine as it enabled patient evaluations without potential exposure to the virus. During the pandemic, an increase utilization of 154% was noted in March of 2020 relative to the same week just one year prior to the pandemic (1).

Prior to the pandemic, there had been marginal interest in expanding the usage of telemedicine in the United States, including in specialities such as pediatric urology. Telemedicine was not widely used by pediatric urologists prior to the COVID-19 pandemic with only a few examples demonstrating its utility for postoperative evaluations (2, 3), prenatal consultations (4), and management of enuresis (5). As in many other specialties, pediatric urologists were forced to rapidly increase usage of this type of patient care due to restrictions linked to the pandemic (6–8). Although prior to the pandemic, multiple studies have demonstrated patient and family satisfaction with utilization of telemedicine (9–11), limited information exists on the perceptions of telemedicine usage after the COVID-19 pandemic. Thus, the purpose of this study was to evaluate the impact of the pandemic on utilization of telemedicine in pediatric urology.

## Methods

A prospective survey was conducted to investigate caregivers’ attitudes toward telemedicine appointments and factors related to telemedicine utilization, the survery can be found in Appendix A. This study gained exempted IRB approval since no protected health information was obtained. The survey was distributed to all primary caregivers of patients less than 18 years of age during either an in-person (IP) or a telemedicine (TM) encounter from July 2021 through October 2021. Since the survey was conducted approximately one year after the onset of the pandemic, patients were offered either IP or TM evaluations with our team. As the pandemic evolved, so did the usage of telemedicine. There were times when only telemedicine visits were offered and later times were it was optional and a specific number were conducted to optimize clinic flow. Caregivers of patients seen *via* telemedicine were administered the survey verbally while caregivers in clinic completed the survey either electronically or verbally depending on their preference. Exclusion criteria included caregivers (1) who declined participation; (2) whose child was greater than 17 years of age, or (3) who did not speak English.

Specific topics queried included patient and caregiver age, experience with telemedicine, and factors potentially influencing caregiver desire to use this modality (Appendix A). Other physical factors queried included distance from provider, community population, available bandwidth, and equipment for performing telemedicine (desktop, laptop, tablet, or smartphone).

Demographic information was characterized by frequencies and percentages. Also, frequency counts and percentages of responses were calculated for each survey item to analyze the results. An open-ended question regarding additional limitations to telemedicine usage was included in the survey, and the responses were independently coded and organized into thematic categories. Frequency of responses to thematic categories was compared between the IP and TM group by Fisher’s exact test. The association between patient’s distance to the clinic and their willingness to use telemedicine was determined with the Wilcoxon rank sum test.

## Results

Two hundred and thirty-nine total patient caregivers of pediatric urology were surveyed: 209 IP and 30 TM. The two groups were comparable in patient and caregiver ages, gender, and ethnicity. The majority of patients were male and Caucasian in both groups (**Table 1**). Of the caregivers surveyed, the majority in both cohorts reported being more likely to use telemedicine now than before the pandemic: IP (125/209, 59.8%) and TM (23/30, 76.7%), with 59/209 of the IP group and 4/30 TM group responding as neutral (**Table 2**). Patients in the TM group were more likely to have used telemedicine prior to the survey as well: 20/30 (66.7%) versus 44/209 (21.1%) (p < 0.001). Furthermore, of those patients who had used telemedicine previously, those in the TM group were more likely to have had multiple visits as compared to the IP group (p = 0.002). Regarding how many times caregivers had previously used telemedicine, the majority of the IP caregivers (29) who had experience using telemedicine had only used it once. Yet, in the TM cohort, 7 caregivers had used telemedicine 2 times before and 6 caregivers had used telemedicine 4 or more times before.

**Table 1:** Frequencies and percentages of responses to demographic information by location of physician visit.

	Visit Setting		
Survey item	In clinic (n=209)	Telemedicine (n=30)	p-value
Child’s age			
0-12 months	48 (23.0)	7 (23.3)	0.268
1-3 years	30 (14.3)	0 (0.0)	
4-7 years	49 (23.4)	8 (26.7)	
8-12 years	53 (25.4)	10 (33.3)	
> 12 years	29 (13.9)	5 (16.7)	
Parent’s age			
18-22 years	11 (5.3)	3 (10.0)	0.184
23-30 years	61 (29.2)	9 (30.0)	
31-40 years	90 (43.0)	16 (53.3)	
> 40 years	47 (22.5)	2 (6.7)	
Child’s sex			
Male	144 (68.9)	18 (60.0)	0.329
Female	65 (31.1)	12 (40.0)	
Race			
Black	30 (14.4)	1 (3.3)	0.369
White	164 (78.5)	29 (96.7)	
Asian American	2 (1.0)	0 (0.0)	
Native American	1 (0.5)	0 (0.0)	
Multiracial	6 (2.8)	0 (0.0)	
Other	6 (2.8)	0 (0.0)	
Ethnicity			
Hispanic/Latino	13 (6.2)	1 (3.3)	0.625
Non-Hispanic	169 (80.9)	27 (90.0)	
Other	27 (12.9)	2 (6.7)	

**Table 2:** Frequencies and percentages of responses to telemedicine survey post COVID-19 by location of physician visit.

	Visit Setting		
Survey item	In clinic (n=209)	Telemedicine (n=30)	p-value
Have you previously used telemedicine?			
Yes	44 (21.4)	20 (66.7)	< 0.001
No	162 (78.6)	10 (33.3)	
How many times have you used telemedicine?			
1 time	29 (65.9)	4 (20.0)	0.002
2 times	6 (13.6)	7 (35.0)	
3 times	1 (2.3)	3 (15.0)	
4 or more	8 (18.2)	6 (30.0)	
Would you participate in a pre-operative consult via telemedicine?			
Yes	114 (54.6)	6 (20.0)	0.668
No	58 (27.7)	6 (20.0)	
Unsure	37 (17.7)	18 (60.0)	
Do you prefer to use telemedicine for your initial visit?			
Yes	50 (23.9)	12 (40.0)	
No	159 (76.1)	18 (60.0)	
Does the severity of your child's case impact whether you will use telemedicine?			
Yes	162 (77.5)	28 (93.3)	
No	47 (22.5)	2 (6.7)	
Are you more likely to participate in telemedicine than before COVID-19?			
Agree	125 (59.8)	23 (76.7)	0.174
Neutral	59 (28.2)	4 (13.3)	
Disagree	25 (12.0)	3 (10.0)	
Would you use telemedicine for a return visit?			
Yes	128 (61.2)	30 (100.0)	< 0.001
No	27 (12.9)	0 (0.0)	
Unsure	54 (25.9)	0 (0.0)	
Yes	44 (21.4)	20 (66.7)	< 0.001

Some categories will not sum to the total N due to missing responses.

When queried on their willingness to participate in a preoperative consult *via* telemedicine, the majority of the IP group (114/209) responded they would be willing to do so, while the majority of the TM group (18/30) responded that they were unsure (**Table 2**). However, the majority of both groups, IP 159/209 and TM 18/30, said that they would not prefer for their initial visit to take place using telemedicine. While the majority of caregivers in both groups indicated that the severity of their child’s condition would impact their likelihood to utilize telemedicine for evaluation in the future [IP (77.5%) versus TM (93.3%], the TM group was significantly greater than the IP group (p = 0.045). Additionally, although the majority of both groups reported that they would use telemedicine for a return visit, there was a higher likelihood for preference for a return visit *via* telemedicine in the TM cohort as compared to the IP group: TM 30/30 vs IP 128/209 (61.2%) p < .001.

Most caregivers in both groups reported that they would utilize telemedicine within 60 miles from the provider, with the highest percentage of the IP group (82/209) indicating that they would use telemedicine within 30 miles of their provider (**Table 3**). Population distribution was comparable in the two cohorts as well, with the majority of both groups living in cities with <10,000 residents. More than 90% of families in the groups combined reported having both a laptop and a cellular phone in their home. More caregivers in the IP group reported having a desktop computer and a tablet in their home than the TM group (41.1% versus 20.0% and 27.3% versus 3.3%, respectively). The most common device among both groups was a cellular phone: IP (174/209) vs TM (28/30). Finally, no difference was identified in home internet bandwidth between cohorts with the majority of both groups, IP (142) and TM (22), responding that they were unaware of their internet bandwidth.

**Table 3:** Frequencies and percentages of responses to factors affecting potential use telemedicine by physician visit location.

	Visit Setting		
Survey item	In clinic (n=209)	Telemedicine (n=30)	p-value
What distance would you need to be from provider to use telemedicine?			
> than 180 miles	11 (5.3)	1 (3.3)	0.568
> than 150 miles	8 (3.8)	3 (10.0)	
> than 120 miles	13 (6.2)	3 (10.0)	
> than 90 miles	23 (11.0)	2 (6.7)	
> than 60 miles	29 (13.9)	6 (20.0)	
> than 30 miles	43 (20.6)	4 (13.3)	
< than 30 miles	82 (39.2)	6 (36.7)	
What is the population of your city of residence?			
< 10,000	73 (34.9)	18 (60.0)	0.15
10,001 – 20,000	26 (12.4)	4 (13.3)	
20,001 – 30,000	24 (11.5)	1 (3.3)	
30,001 – 40,000	23 (11.0)	1 (3.3)	
40,001 – 50,000	10 (4.8)	0 (0.0)	
> 50,000	53 (25.4)	6 (20.0)	
What device do you have to use telemedicine?^a^			
Computer	86 (41.2)	6 (20.0)	0.026
Laptop	161 (94.7)	24 (96.0)	0.784
Tablet	57 (27.3)	1 (3.3)	0.004
Cell phone	174 (83.3)	28 (93.3)	0.186
Other	3 (1.4)	0 (0.0)	0.509
What is your home internet bandwidth?			
< 25 mbps	13 (6.2)	0 (0.0)	0.716
26 – 50 mbps	15 (7.2)	2 (6.7)	
51 – 75 mbps	16 (7.7)	2 (6.7)	
76 – 100 mbps	12 (5.7)	1 (3.3)	
> 100 mbps	11 (5.3)	3 (10.0)	
I don’t know	142 (67.9)	22 (73.3)	

a Values will sum to greater than 100% due to multiple answers per respondent. No respondent reported “no” to all devices.

Responses to the single open-ended question on telemedicine limitations were categorized thematically. The majority of caregivers (120/239) reported no problems (**Table 4**). Of those reporting a limitation, slow bandwidth or cellular connection was the most frequently reported limitation: 15/209 (7.2%) IP and 4/30 (13.3%) TM. The other notable limitation reported was severity of their child’s illness: 12/209 (5.7%) IP and 2/30 (6.7%) TM. Additional limitations included availability of telemedicine appointments, demonstration of their child’s genitalia on video, and technical issues unrelated to wireless connection. No statistical differences in the thematic responses between the IP and TM cohorts were noted.

**Table 4:** Frequencies and percentages of limitations to telemedicine by location of visit.

Limitations^*^	In clinic (n=209)	Telemedicine (n=30)
None or no problems	100 (47.9)	20 (66.7)
Availability of appointments	1 (0.5)	0 (0.0)
Don’t like telemedicine or prefer in person	3 (1.4)	0 (0.0)
Poor broadband or cellular connection	15 (7.2)	4 (13.3)
Uncertain or lack of exposure to telemedicine	4 (1.9)	0 (0.0)
Decision to use based upon severity of the child’s illness	12 (5.7)	2 (6.7)
Unwilling to show genitalia on video	1 (0.5)	0 (0.0)
Other technical issues unrelated to broadband or cellular connection	2 (1.0)	0 (0.0)
Miscellaneous	1 (0.5)	1 (3.3)
No answer	70 (33.5)	3 (10.0)

^*^There was no significant difference in the frequency of responses to limitations between location of visit (p=.113).

## Discussion

In parallel to the recent increased utilization of telemedicine during the pandemic, there also has been expanded research effort evaluating the utilization of this tool in pediatric urology for care (6–8, 12, 13). The majority of these studies are retrospective in nature and examine the impact on telemedicine usage in volume, patient/family satisfaction, and tips on utilization of telemedicine. One meta-analysis was performed on the available literature on telemedicine in pediatric urology with 17 studies meeting the inclusion criteria (13). Four studies included a comparision or control group, but none were randomized. All papers support expanded telemedicine in pediatric urology because of improved access, patient and family satisfaction, and equivalent outcomes. However, no information has specifically addressed the impact on caregiver perceptions of this technology such as the current study has undertaken.

The most predominant finding of the preferences identified is that caregivers of children with pediatric urological diseases are more likely to use telemedicine now than before the pandemic (IP 59% vs TM 76.7%). This is the first such data demonstrating increased interest in telemedicine utilization in pediatric urology after the pandemic and is evident regardless of prior experience with telemedicine or the methodology for the current patient evaluation. Although the number of observations in the two cohorts are low, inclusion of an in-person cohort reduces inherent bias brought about by caregivers currently being interviewed through a telemedicine platform. Lastly, these results are consistent with the overall patient and family satisfaction with telemedicine utilization noted by other studies in this specialty since the pandemic began.

Other information surveyed included caregiver preferences related to variable telemedicine encounter categories. For instance, caregivers were queried on whether they would choose a preoperative evaluation or an initial evaluation through telemedicine. Although the majority in both cohorts reported preferring an in-person evaluation for the initial visit, no statistically significant findings were observed for either group for these two encounter types. However, both groups indicated that the severity of their child’s illness would impact their willingness to use telemedicine. Individuals in the telemedicine cohort placed greater value upon the severity of illness (93.3%) on their willingness to use this technology relative to the in-person group (77.5%).

Survey responses also indicated that caregivers were more likely to use telemedicine for their visit if they were within 60 miles of their provider. Initially, it was hypothesized that patients further from their provider would be more inclined to use telemedicine due to the reductions in travel time and cost for in-person evaluation. Research at the University of Virginia demonstrated a median reduction of 2.25 hours of travel time for patients evaluated with telemedicine (8). Other studies previously demonstrated that telemedicine reduces the travel and time required for treatment as well (2, 3, 7). Based on these findings, it appears that patient families consider and prioritize various factors such as complexity and perhaps the nature of the evaluation (i.e. preoperative or initial consultation) when making a decision upon whether to engage in telemedicine when given the option. Further study is needed to better understand how these factors impact family decision-making on telemedicine evaluation.

While there was some variation between the groups in regard to specific devices, the majority of both groups reported having two devices with telemedicine capabilities, specifically a computer and a cellular phone. More families in the in-person group reported having a desktop computer and tablet in their home compared to the telemedicine group. Additionally, most individuals across both groups reported being uncertain about the internet or cellular connectivity, and, therefore, no conclusions can be drawn about the internet capabilities between the groups or the impact of this factor upon the other perspectives submitted. The fact that patients have access to multiple devices capable of telemedicine within a household suggests that patients and providers have the necessary equipment for interfacing with telemedicine in the future. However, a recent postoperative study in pediatric urology indicated that 14% of patients were unable to connect at the time of their telemedicine visit (3). Another large academic center in Wisconsin (137 846 scheduled video visits) demonstrated slow high-speed internet connection as one factor that increases the likelihood of conversion to a telephone visit (14). The internet connection also was the most commonly reported problem in the open-ended telemedicine limitations question in the present study. The full impact of slow bandwidth or cellular connection upon telemedicine utilization is not know and requires further study. However, the results of the current study suggest that the limitation is not due to availability of devices for telemedicine utilization.

The most significant limitation to this study was the small patient numbers collected especially in the telemedicine cohort despite the prospective nature of the analysis. Also, the study was conducted midway through the pandemic leading to the variation in sample size (209 IP vs. 30 TM) relative to early in the pandemic. A greater number of participants might help better understand caregiver preferences including the perspectives on preoperative and initial consultations. Lastly, lack of inclusion of associated disease-specific data for those surveyed is a limitation as well. Additional information might be gleaned by further study of specific disease processes of varying severities to better understand the impact of severity of disease on utilization of telemedicine.

## Conclusions

The COVID-19 pandemic has increased the usage of telemedicine in various specialties, including pediatric urology. Caregivers of pediatric urology patients are more inclined to use telemedicine than prior to the pandemic, and have the technology to do so from home. Factors such as the severity of the urological issue and the distance the patient is from the provider impact caregivers willingness to use telemedicine. The accessibility and ease that is associated with telemedicine usage will likely continue to increase its usage among patients and providers as we transition out of the pandemic.

## Data availability statement

The original contributions presented in the study are included in the article/**Supplementary material**. Further inquiries can be directed to the corresponding author.

## Author contributions

TB, SC, BS contributed to the conception and design of the study. TB administered the survey analyzed in this study. BS performed the statistical analysis. TB wrote the first draft of this manuscript. SC wrote sections of this manuscript. All authors contributed to manuscript revision, read, and approved the submitted version.

## References

[1] Koonin L Hoots B Tsang C Leroy Z Farris K Jolly BT. Trends in the use of telehealth during the emergence of the COVID-19 pandemic — united states, January–march 2020. MMWR Morb Mortal Wkly Rep (2020) 69:1595–9. doi: 10.15585/mmwr.mm6943a3 PMC7641006 33119561

[2] Canon S Shera A Patel A Zamilpa I Paddack J Fisher PL. Telemedicine for post-operative urologic care in children: A pilot study. J Telemed Telecare. (2014) 20(8):427–30. doi: 10.1177/1357633X14555610 25316038

[3] Finkelstein JB Cahill D Young K Humphrey K Campbell J Schumann C. Telemedicine for pediatric urological postoperative care is safe, convenient and economical. J Urol. (2020) 204(1):144–8. doi: 10.1097/JU.0000000000000750 31951495

[4] Rabie NZ Canon S Patel A Zamilpa I Magann EF Higley J. Prenatal diagnosis and telemedicine consultation of fetal urologic disorders. J Telemed Telecare (2015) 21. doi: 10.1177/1357633X15595556 26199277

[5] Smith E Cline J Patel A Zamilpa I Canon S. Telemedicine versus traditional for follow-up evaluation of enuresis. Telemedicine e-Health. (2021) 27(2):213–7. doi: 10.1089/tmj.2019.0297 32539570

[6] Finkelstein JB Nelson CP Estrada CR. Ramping up telemedicine in pediatric urology- tips for using a new modality. J Pediatr Urol (2020) 16(3):288–9. doi: 10.1016/j.jpurol.2020.04.010 32327290

[7] Gan Z Lee SY Weiss DA van Batavia J Siu S Frazier J. Single institution experience with telemedicine for pediatric urology outpatient visits: Adapting to COVID-19 restrictions, patient satisfaction, and future utilization. J Pediatr Urol (2021) 17(4):480.e1–7. doi: 10.1016/j.jpurol.2021.05.012 PMC8491551 34078574

[8] Winkelman AJ Beller HL Morgan KE Corbett ST Leroy SV Noona SW. Benefits and barriers to pediatric tele-urology during the COVID-19 pandemic. J Pediatr Urol (2020) 16(6):840.e1–6. doi: 10.1016/j.jpurol.2020.09.028 PMC7543732 33077389

[9] Chrapah S Becevic M Washington KT Sheets LR Wallach E Chitima R. Patient and provider satisfaction with pediatric urology telemedicine clinic. J Patient Experience (2021) 8:237437352097573. doi: 10.1177/2374373520975734 PMC8205389 34179351

[10] Miller GG Levesque K. Telehealth provides effective pediatric surgery care to remote locations. J Pediatr Surg (2002) 37(5):752. doi: 10.1053/jpsu.2002.32270 11987093

[11] Shivji S Metcalfe P Khan A. Pediatric surgery telehealth: Patient and clinician satisfaction. Pediatr Surg Int (2011) 27(5):523. doi: 10.1007/s00383-010-2823-y 21243367

[12] Holzman SA Davis-Dao CA Khoury AE Fortier MA N Kain Z. Telemedicine and patient satisfaction during the COVID-19 pandemic: A case-control study of outpatient pediatric urology patients. J Child Health Care (2021) 6:13674935211058272. doi: 10.1177/13674935211058272 34865548

[13] Pettit S Young E Jung I. Systematic review of telemedicine in pediatric urology. J Pediatr Urol (2022) 18(1):17–22. doi: 10.1016/j.jpurol.2021.09.024 34642111

[14] Crotty BH Hyun N Polovneff A Dong Y Decker MC Mortensen N. Analysis of clinician and patient factors and completion of telemedicine appointments using video. JAMA Netw Open (2021) 4(11):e2132917. doi: 10.1001/jamanetworkopen.2021.32917 34735013 PMC8569484

