# Supplementary material for: Telemedicine preferences in pediatric urology following the COVID-19 pandemic: A caregiver survey
Source: Front Urol. 2023 Feb 13;3:994540. doi: 10.3389/fruro.2023.994540 (PMC12327306; doi:10.3389/fruro.2023.994540)
Supplement: Supplementary file 1 [file DataSheet_1.docx]

***Appendix A***

**Post COVID-19 Telemedicine Preferences Survey.**

**About your child**

**Clinic Visit location:**

Clinic

Telehealth

**Your child’s diagnosis/ reason for visit?**

**Your child’s age:**

0-6months

6-12 months

1-2 years

2-3 years

3-4 years

4-8 years

8-12 years

>12 years

**Your child’s sex:**

Male

Female

Other

**About you**

**Your age?**

18-22 years

22-30 years

30-40 years

>40 years

**Your race:**

White

Black/African American

Asian American

Native American/Alaska Native

Native Hawaiian/Other Pacific Islander

Multi-racial

Other

**Your ethnicity:**

Non-Hispanic

Hispanic/Latino

Other

**Have you previously used telemedicine for your child’s health care appointment?**

No Yes

**How many times?**

0

1

2

3

4 or more

**If you do use telemedicine, approximately how far away from your provider do you live?**

1. Less than 30 miles from the provider
2. Greater than 30 miles from the provider
3. Greater than 60 miles from the provider
4. Greater than 90 miles from the provider
5. Greater than 120 miles from the provider
6. Greater than 150 miles from the provider
7. Greater than 180 miles from the provider

**Is there a certain distance between you and your provider that makes you more inclined to participate in a telemedicine visit rather than physically come in to the clinic?**

1. Less than 30 miles from the clinic
2. Greater than 30 miles from the clinic
3. Greater than 60 miles from the clinic
4. Greater than 90 miles from the clinic
5. Greater than 120 miles from the clinic
6. Greater than 150 miles from the clinic
7. Greater than 180 miles from the clinic

**Would you participate in a return visit using telemedicine?**

No Yes Unsure

**Would you participate in a pre-operation consultation visit using telemedicine?**

No Yes Unsure

**Would you prefer using telemedicine for your initial visit?**

No Yes

**Would the severity of the problem your child is being seen for impact whether you used telemedicine for the initial visit?**

No Yes

**What type of device do you use for telemedicine visits? (mark all that apply)**

Computer Tablet Cell phone Other, explain

**What type of bandwidth do you have at your place of residence?**

_

1. 1-25 Mbps
2. 26-50 Mbps
3. 51-75 Mbps
4. 76-100 Mbps
5. 101+ Mbps
6. I don’t know

**What is the population of the city where you currently live?**

1. Less than 10,000
2. 10,001-20,000
3. 20,001-30,000
4. 30,001-40,000
5. 40,001-50,000
6. 50,001+

**Do you have a computer in your home?**

No Yes

**If yes, do you have a laptop computer?**

No Yes

**Are you more likely to participate in telemedicine visits than before COVID-19?**

Strongly agree Agree Neither agree nor disagree Disagree Strongly disagree

**Are there any limitations to your telemedicine usage?** (Leave this as an open-ended option)
